# Cycling in one of the most polluted cities in the world: Exposure to noise and air pollution and potential adverse health impacts in Delhi

**DOI:** 10.1186/s12942-021-00272-2

**Published:** 2021-04-30

**Authors:** Philippe Apparicio, Jérémy Gelb, Vincent Jarry, Élaine Lesage-Mann

**Affiliations:** Environmental Equity Laboratory, Institut National de La Recherche Scientifique, 385 rue Sherbrooke Est, Montréal, Québec H2X 1E3 Canada

**Keywords:** Cycling, Exposure, Inhalation dose, Noise, Air pollution, Delhi

## Abstract

**Background:**

In India, many cities struggle with extreme levels of air pollution and noise. Delhi, in particular, has the notorious reputation of being one of the most polluted cities in the world. Cyclists constitute a particularly exposed population, since they cycle among motor vehicles without any protection. This paper modeled the cyclists’ exposure to nitrogen dioxide (NO_2_) and noise in Delhi, India.

**Methods:**

Using primary data collected on 1,229 kms of roads in Delhi, Generalized Additive Mixed Models with Auto-Regressive terms (GAMMAR) are constructed for noise exposure, NO_2_ exposure and NO_2_ inhalation doses.

**Results:**

Results show that cyclists are exposed to 47 µg/m^3^ of NO_2_ and 3.3 dB(A) more when cycling on a primary road than on a residential street. Using WHO guideline values for noise and air pollution, we assessed how many minutes of inhaling doses and noise doses become potentially harmful to cyclists’ health in Delhi. Such thresholds are quickly exceeded: after cycling one hour in an area with moderate predicted values of noise and air pollution, the noise dose and inhaled dose of NO_2_ will reach 212% and 403 µg on residential streets, and 459% and 482 µg on primary roads, respectively.

**Conclusion:**

Policy makers should take these results into account to minimize cyclists’ exposure, especially for the most deprived people.

**Supplementary Information:**

The online version contains supplementary material available at 10.1186/s12942-021-00272-2.

## Background

Cycling is again becoming an increasingly popular mode of transportation in many Global North cities again. Some authors even refer to a “bicycle renaissance” [1]. After several decades of car-oriented planning, planners of Global North cities today consider cycling as a sustainable means of transportation that helps to reduce greenhouse gases, road congestion and road traffic noise [2]. In that respect, numerous cities are investing massively in cycling infrastructure to get commuters out of cars and increase the modal share of cycling. These include such measures as extending the cycling network, and establishing bike-sharing systems and a physical separation from motor vehicles [1]. As a result, cycling is tending to become safer in northern cities. However, the picture is quite different in numerous cities of the Global South, including Indian cities. Various factors make cycling riskier, such as little or no cycling infrastructure, the fast-growing car fleet and road traffic, and worrisome noise and air pollution levels [3]. Consequently, cyclists’ exposure to air and noise pollution becomes a major issue in Global South cities, especially in India. Yet this question has been little explored in cities in the Global South, unlike those in the North.

### Cycling in Delhi

**Fig. 1:**
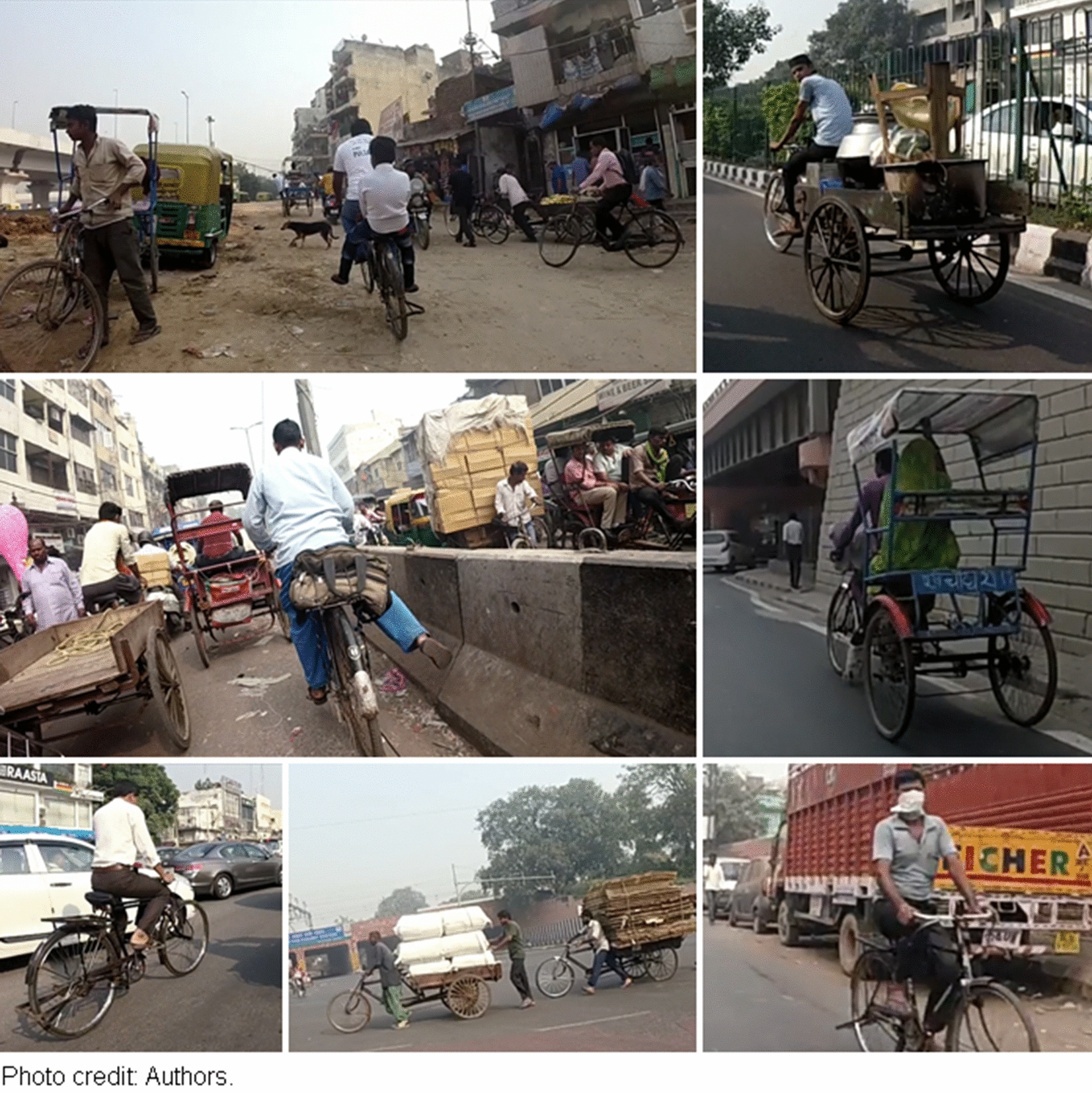
Cyclists in Delhi

Few authors have studied cycling in India and particularly in Delhi. Tiwari, Jain and others have carried out pioneering work in this field (e.g. [4], [5–8]). According to these authors, cycling was once a very popular means of transportation in Delhi: in 1980, almost 18% of all trips were by bicycle. However, as incomes and motorization levels increased, cycling faced a sharp decline: in 2005, less than 5% of all trips were by bicycle [4]. This is analogous to what happened in many northern cities between the 1950s and the 1980s, when government efforts were primarily focused on designing efficient infrastructure for motorized vehicles [9]. People still cycling in Delhi generally have few options: their choice is constrained by insufficient economic resources to access any motorized modes of transportation [6]. Non-motorized transport, especially walking and cycle rickshaws, is often used to access public transit and thus increase its catchment area [4]. According to Ravi [10], there are approximately 600,000 rickshaw drivers in Delhi. Moreover, bicycles are used by many low-wage workers in Delhi (e.g. rickshaws and delivery of many goods) [8], as illustrated in Fig. 1. Nonetheless, there is very little cycling infrastructure dedicated to cyclists, resulting in high rates of collisions, high level of exposure to air pollution and noise [4, 6]. Cyclists in Delhi are overrepresented in traffic fatalities: they account for 10% of deaths, while only constituting less than 5% of all trips [4]. In short, in Indian cities such as Delhi, cycling is clearly associated with poverty, informal and precarious workers, and hostile conditions [3].

### Cyclists’ exposure to air pollution and noise

The individual and collective advantages of cycling in urban areas are now well known [11]. Individually, cycling has multiple benefits for health and well-being (improving cardiovascular fitness, and reducing the risk of chronic diseases, being overweight and obese, and of all causes of mortality) [11–13]. Collectively, travelling by bicycle has positive effects on reducing traffic congestion, greenhouse gas emissions, and noise, but also on reducing health care costs [11, 14]. Despite these widely documented benefits, cycling in urban areas is associated with health and safety risks owing to potentially high levels of traffic density and exposure to air pollution and road traffic noise [12]. It is well-known that prolonged exposure to high levels of air pollution and road traffic noise may be harmful to human health and well-being (e.g. increasing the risk of respiratory and cardiovascular diseases, certain types of cancer, annoyance and stress) [15, 16].

Because of their higher levels of ventilation rates, cyclists inhale more air pollutants than pedestrians, public transit users and, most notably, motorists. In a recent systematic review, Cepeda et al. concluded that, on average, motorists inhale only 16% of the total dose of pollutants inhaled by active commuters [17]. Other authors, comparing the individual exposure to noise by mode of transportation, have demonstrated that cyclists′ levels of exposure to noise are significantly greater than motorists [18–20]. For instance, a difference of 2 dB(A) was found in Montreal [18] versus 4 dB(A) in Thessaloniki and 5 dB(A) in Helsinki [20].

Despite these higher exposure levels for cyclists, numerous studies have concluded that health benefits outweigh the risks incurred [12, 21–23]. However, most of these studies have been conducted in Global North cities where air and noise pollution levels are much lower than in Global South cities [23, 24]. In the same vein, Tainio et al. [21] estimated that harms caused by air pollution would outweigh health benefits after 90 min of cycling in areas with PM_2.5_ concentration of 100 µg/m^3^. Using the World Health Organization Ambient Air Pollution Database, they found this worrisome situation applies to only 1% of 1,622 cities around the world. In Delhi, annual average concentration of PM_2.5_ reached 116, 123, 143 ug/m^3^ in 2014, 2015 and 2016, respectively. That shows air pollution in Delhi could be a major health concern for cyclists.

Finally, over the past two decades, numerous studies have shown that traffic flows, proximity to motor vehicle traffic and the type of road taken by the cyclist can all have a significant impact on the exposure to air pollution and noise. Yet, there again, studies have generally focused on Global North cities [25].

### Cyclists’ exposure in Delhi

Studies of cyclists’ exposure in Global South cities are very rare. In Delhi specifically, Goel et al. [26] compared particulate matter (PM_2.5_) exposure in 11 transport microenvironments. With a 41-day data collection, they measured mean exposure levels of 347 µg/m^3^ in January and 285 µg/m^3^ in February 2014. Since cyclists have a higher breathing rate, the authors found that they inhale nine times more pollutants per kilometre than air-conditioned car occupants. Even more shockingly, cycling for an hour during the morning peak period leads to inhaling 40% more PM_2.5_ than when cycling for an entire day in a Global North city where PM_2.5_ levels are between 10 and 20 µg/m^3^.

Saksena et al. [27] offer a more general study on daily exposure (indoor, outdoor and in-vehicle) to particulate matter (all sizes) and carbon monoxide (CO) in Delhi. Although they measured exposure for two-wheelers (e.g. scooters and motorcycles), we can assume that their exposure levels might be similar to those of cyclists. Thus, two-wheeler’s exposure (2860 µg/m^3^ for PM and 19 ppm for CO) is far higher than car occupants’ exposure (370 µg/m^3^ for PM and 10 ppm for CO) and that of bus users (800 µg/m^3^ for PM and 8 ppm for CO). Regardless of the mode of transportation used, the particulate matter dose inhaled by students and workers in transport represents 12 to 20% of their total daily dose. For CO, transport represents 50 to 60% of daily inhaled dose for students, versus 32 to 38% for workers.

To our knowledge, no study has yet investigated cyclists’ exposure to noise in Delhi. However, a recent investigation measured this in Nagpur City (India) at 700 sampling locations [28]. On major roads, they reported an average noise value of 90.0 dB(A) (*L*_eq_), versus 84.1 on minor roads. They identified traffic congestion as the main source of noise during their data collection. This corroborates the results of Kumar et al. [19] who found a mean noise exposure levels of 84 dB(A) in a bus, 89 dB(A) in a rickshaw and 72 dB(A) in a car in Delhi. More recently, Akhtar et al. [29] measured and mapped noise for three predetermined sites (one residential area and two commercial areas) in Delhi. They observed that noise levels greatly exceeded permissible limits, with mean levels at 75 dB(A) and hourly peaks at 80 dB(A) for the three sites. Unsurprisingly, road traffic appears to be the main source of noise. The highest levels, recorded directly on the road, reach more than 80 dB(A).

### Study objectives

To the best our knowledge, in Delhi, no study has measured cyclists’ exposure to road traffic noise and traffic-related air pollution simultaneously. To fill this gap, the purpose of this study is twofold. The main objective is to identify factors, which significantly influence cyclists’ exposure to air pollution (NO_2_) and road traffic noise, and inhaled dose of NO_2_ throughout Delhi, paying particular attention to the type of road taken by the cyclist. It is worth noting that transportation is that main source of NO_2_ whereas PM_2.5_ is caused more by cooking combustion and crop residue burning, especially in Delhi [30, 31]. Our second objective is to assess after how many minutes total inhaled dose of NO_2_ and total noise dose in Delhi become potentially harmful to cyclists’ health, according to the guidelines of the World Health Organization (WHO).

## Data and methods

### Study design and approach

**Fig. 2:**
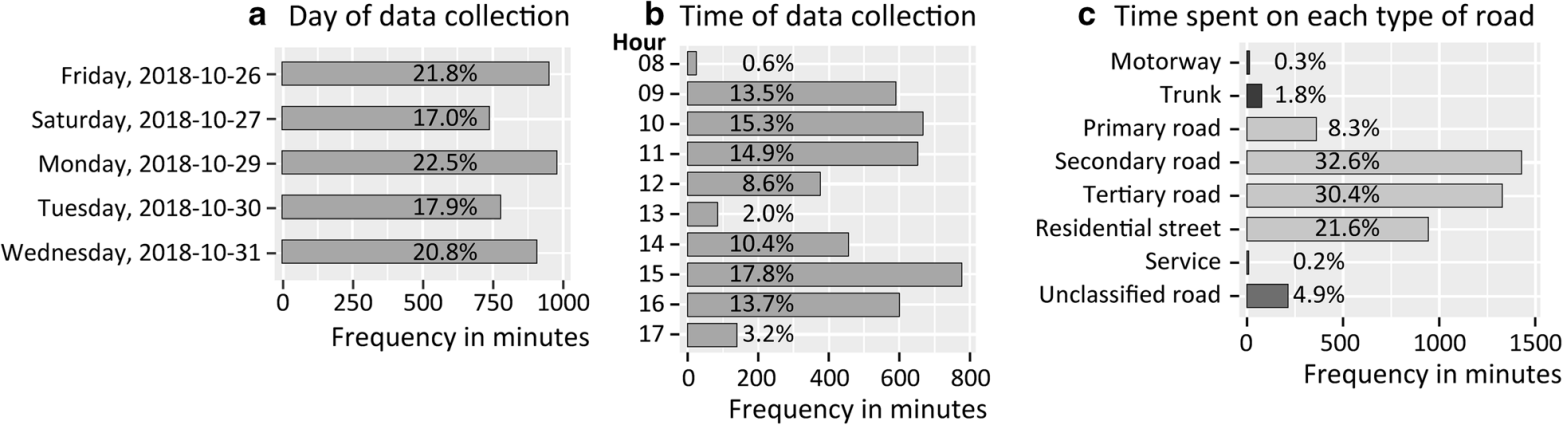
Distribution of the collected data by day, time and road type

Two graduate students and one professor in Urban Studies (the first three authors of the manuscript; male; age: 23, 24, 46; in good shape and with high urban cycling experience) were involved in the data collection during five dry days (26–27, 29–31, October 2018) (Fig. 2a). At this time of year, intense crop residue burning occurs both in Delhi and in northern adjacent regions, leading to high levels of air pollutants, especially particulate matter [30]. However, in an inventory of air pollution emissions in Delhi, Guttikunda et al. [31] estimated that waste burning in general is only responsible for 1% of NO_x_ emissions, which are most closely related to vehicles.

**Fig. 3:**
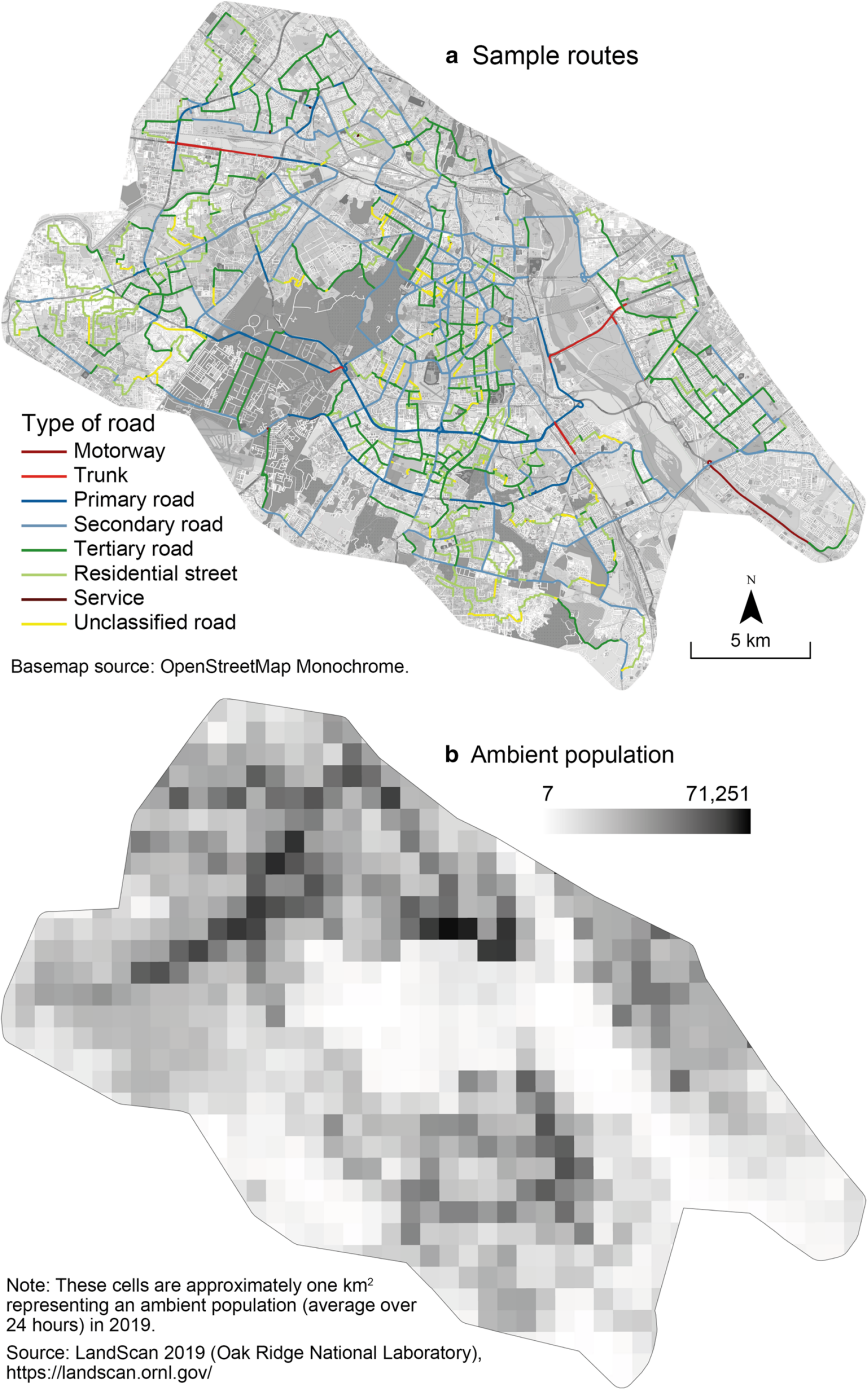
Study area and sample routes

During the data collection, each participant cycled between seventy and one hundred kilometres per day. In total, 1,229 kms (72 h) were travelled throughout Delhi. The routes were previously defined using GoogleMyMaps in order to maximize the coverage of Delhi (Fig. 3a) and the diversity of road types taken by the cyclist (Fig. 2c), following an extensive data collection design [32]. Each participant cycled specific routes that were never similar, between 08:00 a.m. and 06:00 p.m. (Fig. 2b). There are fewer observations between 12:00 p.m. and 02:00 p.m. because of the lunch break and the recharging of device batteries for data collection. In terms of road diversity, we rarely cycled on motorways, trunks, service and unclassified roads (13, 77, 9 and 214 min, respectively), but mostly on secondary and tertiary roads, and residential streets (1,426, 1,326 and 942 min, respectively). Unlike other data collections by the research team in European and North American cities, three local guides were hired for safety reasons; each of them was paired with a cyclist. In the field, the participants and the local guides followed the determined routes on a cellphone attached to the handlebars. The local guides were instructed to operate their scooter about 20 m in front of the cyclist. Due to high levels of traffic density, air pollution and road traffic noise in Delhi, we can assume that following the guide’s scooter had a very weak impact on exposure levels recorded by the cyclist.

### Measurements of individual air pollution and noise exposure

Seven types of devices were used during the data collection: (1) Brüel and Kjaer personal noise dosimeters (Type 4448—class 1, Narum, Denmark), (2) low cost air quality monitors, Aeroqual Series 500 monitors (Auckland, New Zealand), (3) Hexoskin Smart T-shirts (Montreal, Canada), (4) Garmin GPS watches (Forerunner 920 XT, Olathe, KA, USA), (5) Garmin action cameras (VIRB XE, Olathe, KA, USA), (6) cellphones, and (7) Techno Plus™ Masks with Techno™ Filter (Egham, UK). We thus synchronized the first five devices’ clocks every morning during the data collection.

First, the Brüel and Kjaer device, fixed on the cyclist’s right shoulder, records the average decibel level (dB(A)) every minute (*L*_Aeq,1 min_). As recommended by the manufacturer, the three personal noise dosimeters were calibrated once a day using the sound calibrator type 4231. Their temporal resolution of one minute is sufficiently detailed considering that, with a mean speed of 15 km/h, a cyclist can ride only 250 m. More specifically, the 3 dB(A) exchange rate is used, which means that an increase of 3 dB(A) doubles the noise intensity.

Second, the Aeroqual monitor fixed on the cyclist’s left shoulder has two sensors—a nitrogen dioxide (NO_2_) sensor and temperature and humidity sensor—that record the average NO_2_ value (μg/m^3^), the temperature in degrees Celsius, and the percentage of relative humidity every minute. According to the Aeroqual supplier's product information, the NO_2_ sensor has the following characteristics: range (0–1 ppm), minimum detection (0.005 ppm), accuracy of factory calibration (< ± 0.02 ppm 0–0.2 ppm; <  ± 10% 0.2–1 ppm), and resolution (0.001 ppm). The NO_2_ sensors used were pre-calibrated by the supplier before the data collection.

Third, each participant wore a Hexoskin Smart T-shirt [33] as it assesses the cyclist’s the heart rate, breathing rate and minute ventilation [34–36]. Next, the potential inhaled dose (I) in micrograms of NO_2_ per minute was obtained by multiplying the minute ventilation (VE) in litres per minute obtained through the Hexoskin by the NO_2_ concentration value in µg/m^3^ acquired via the Aeroqual sensor [18, 34] as follows:

1 $$I=\left(VE\times 0.001\right)\times {NO}_{2}$$

The city of Delhi is well-known for its high levels of air pollutants. As a precautionary measure, each cyclist wore a Techno Plus™ Mask with Techno™ Filter during the cycling activity [37]. Respirators have only an infinitesimal impact on the cyclist’s ventilation rate when they respect the overall adjustment factor (user seal check, normal and deep breath fit testing procedures, etc.) to allow better breathing while blocking various pollutants [38, 39].

### GIS data preparation and analyses

**Fig. 4:**
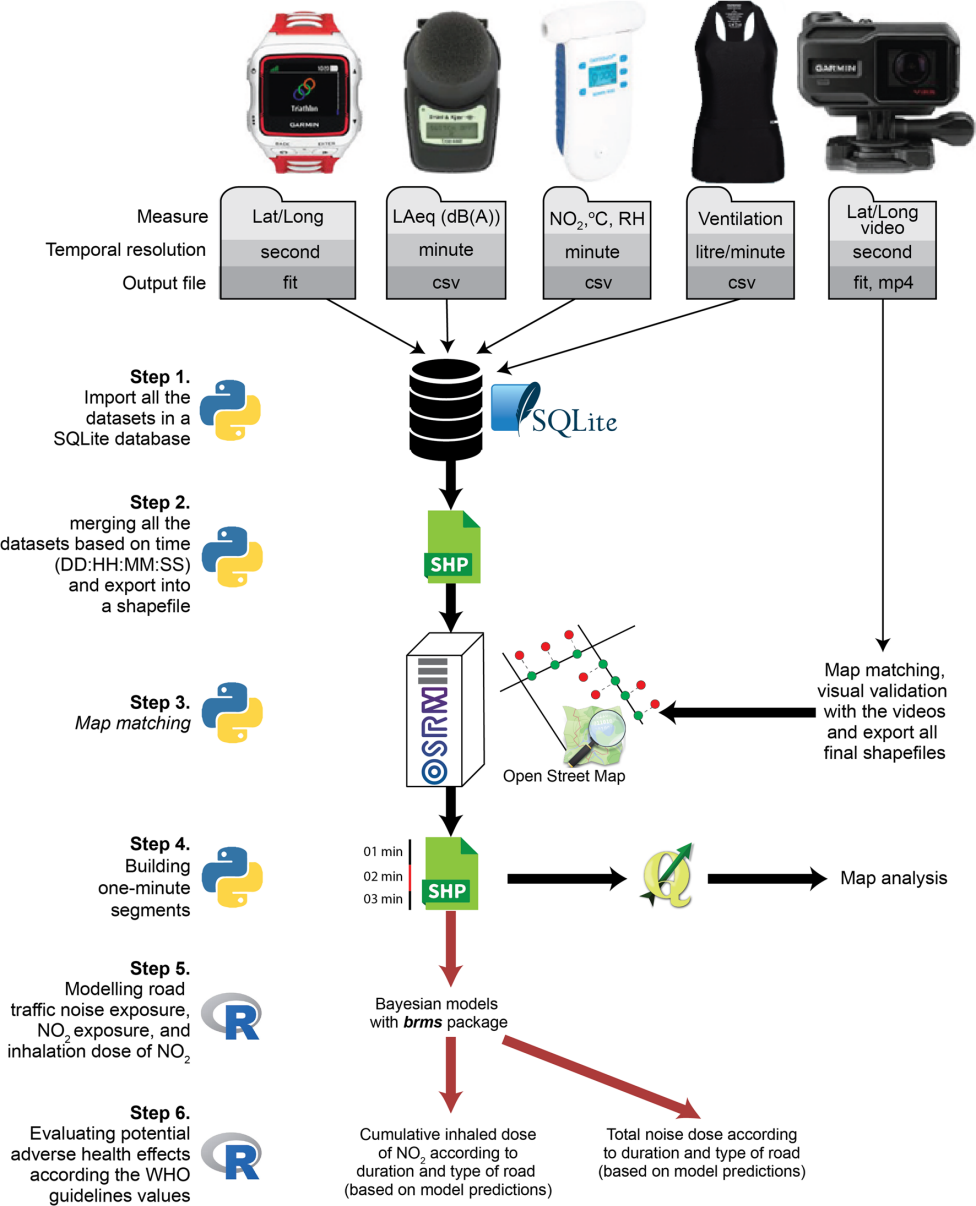
GIS Data preparation and analyses

The data preparation and analysis are illustrated in Fig. 4. All the datasets collected by the devices (Garmin watch, noise dosimeter and air quality monitor, and Hexoskin T-shirt) are imported in a SQLite database using a Python script (Step 1). The Garmin watch records the geographic coordinates each second (the finest temporal resolution among the devices). Consequently, these data tables are merged based on this timestamp (DD:HH:MM:SS), and then exported as a point shapefile for each sample route (Step 2). These GPS points are map-matched on the Open Street Map (OSM) network [40, 41] using the OSRM algorithm [42]; the videos recorded by the Garmin Virb action cameras are used to manually validate the map-matching process (Step 3). The OSM network was selected for two main reasons. First, it facilitates comparisons with other studies as recommended by Gelb and Apparicio [43]. Second, the road type (highway tag) and the road geometries are known to be consistent, considering the structuring role of the road network in the community mapping process [44].

Next, a line shapefile of each trip was obtained and then divided into one-minute segments for which the average noise exposure (*L*_Aeq,1 min_), average NO_2_ exposure (μg/m^3^) and NO_2_ inhaled dose (μg) levels are calculated (Step 4). Finally, these line shapefiles are imported into R to conduct statistical analyses and to evaluate potential adverse health effects, as described in the next two subsections (steps 5 and 6).

### Statistical analyses

All statistical analyses were performed using R for statistical computing software version 3.6.1 [45].

#### Modelling road traffic noise exposure, air pollution exposure and inhalation

To achieve the first objective, three Bayesian models were constructed using the *brms* package [46, 47], in which the dependent variables are the level of noise exposure, the level of NO_2_ exposure and the inhaled dose of NO_2_, and the observations are the one-minute segments (*N* = 4,157, 4,368 and 4,368, respectively). The models proposed here are largely based on recent studies [43, 48, 49]: GAMMAR models (generalized additive mixed model with an autoregressive term) with a student distribution for the dependent variable [50]. Consequently, four types of terms were introduced into each model: random effects terms, non-linear terms (i.e. splines), an autoregressive term, and fixed linear terms.

As the road traffic noise and air pollution could vary according to the day of the week, this introduced into the three models as a random effect. The participant was also introduced as a random effect for two main reasons. First, individuals' measures of noise and air pollution exposure (NO_2_) may vary systematically with the dosimeter and Aeroqual sensor assigned to each participant during the entire data collection period. This is especially applicable for Aeroqual sensors due to the accuracy of factory calibration, as mentioned previously. Second, the inhaled dose of NO_2_ may vary according to the participants' physiological characteristics. At an equal level of intensity of physical exercise and NO_2_ exposure, the participants do not have the same ventilation rates and, thus, their inhaled doses of pollutant may vary (due to their different ages and physical conditions, or even their stress levels when they cycle in the midst of heavy traffic).

As done previously [43, 48, 49], to take the temporal and spatial variability into account, the time of day (number of minutes passed since 08:00) and the geographic coordinates were introduced as non-linear terms (i.e., splines). Therefore, we expected to observe peaks of exposure and inhalation during the morning and evening rush hours, and in the most densely populated areas of Delhi. We introduced a moving average term (MA = 3) to control the temporal autocorrelation because consecutive observations are more likely to be similar than observations selected randomly.

Next, the number of intersections crossed, speed (km/h), slope (%) and time spent in minutes on six types of road were introduced as fixed effects (1. trunk or motorway, 2. primary road, 3. secondary road, 4. tertiary road, 5. residential street, 6. unclassified or service road). Note that trunk and motorway, and unclassified and service roads were merged into two categories due to their similarity and the small number of samples on motorway (n = 13) and service roads (n = 9). Unclassified roads correspond to segments of the OSM network with a missing value in the tag highway or segment with the lowest importance in the road network. This category includes a diversity of minor roads supporting local traffic, which is why it was merged with the service roads.

Overall, we expected that exposure and inhalation levels would decrease from a trunk or a motorway to a residential street. Also, we expected speed and slope to increase ventilation rates, and thereby NO_2_ inhaled dose. Finally, two covariates describing the weather conditions were added to the NO_2_ exposure and inhaled dose models: humidity (measured by the Aeroqual sensor) and wind speed (data for each half-hour available for the international airport station). Temperature was discarded because of its strong correlation with humidity and time.

We fitted our three Bayesian models using four chains, each with 4,000 iterations, where the first 1,000 were used as a warm-up for sampling [46]. The used priors are described in the Supplementary Material.

#### Evaluating potential adverse health effects

Regardless of the city, it is very difficult to determine precisely whether the levels of exposure to air pollution and road traffic noise are harmful to cyclists’ health. Here, we propose a simple assessment based on the guideline values defined by the World Health Organization (WHO).

For short-term exposure to nitrogen dioxide, the WHO [51] recommends the guideline value of 200 µg/m^3^ for one hour, as exposure to this pollutant above this level causes “significant inflammation of the airways.” But no information is provided on the levels of physical activity or ventilation rates considered safe above this concentration level. Of course, the ventilation rates vary according to sex, age, body weight and activity level. According to the United States Environmental Protection Agency [52], the minute ventilation (VE) for an average male adult is estimated at 13 L/min with a very low physical activity level (walking at 3.6 km/h), 19 L/min (walking at 4.8 km/h), 30 L/min for an easy cycling activity and finally, 40 L/min for moderate cycling activity at 13 mph (21.9 km/h). However, it is relatively difficult to reach this average speed in urban areas. Several authors regularly suggest a value of 16 km/h [53–56]. In addition, during the data collection in Delhi, the participants cycled at an average speed of 17 km/h. Consequently, we propose to retain two ventilation rate values: 15 L/min and 35 L/min for a pedestrian and a cyclist in an urban environment. By multiplying the WHO’s guideline value of 200 µg/m^3^ for the NO_2_ by these two selected ventilation rates in m^3^ (VE*0.001) and the duration (60 min), we can estimate a dose threshold above which additional exposure would exceed the WHO guideline and, consequently, become potentially harmful. For one hour, we obtained 180 and 420 µg of NO_2_ for a pedestrian and a cyclist, respectively. The threshold value for the pedestrian will be used for comparison with the cyclist. It is worth noting that the 420 µg value is a very conservative estimate. Indeed, for a given NO_2_ concentration level (200 µg/m^3^), the health risks would be higher for a cyclist inhaling 420 µg over one hour compared to a pedestrian only inhaling 180 µg. Incidentally, during smog episodes, WHO [57] recommends reducing physical activity outdoors, thereby minimizing the inhalation of air pollutants. Finally, based on the results of the GAMMAR model for the inhaled dose of NO_2_, we can predict how many minutes it takes to reach the two cumulative intake risk values (180 and 420 µg) according to the type of road taken by a cyclist in Delhi during the period of our data collection.

For the road traffic noise, the WHO reports “the 5% relevant risk increase occurs at a noise level of 59.3 dB *L*_den_ for the incidence of ischaemic heart disease” [58]. However, we cannot directly compare a one-minute mean exposure (*L*_Aeq,1 min_) and daily mean exposure (*L*_den_). Therefore, we propose to compare these values in terms of doses. According to Berger EH [59], the noise dose is the cumulative exposure to noise over time. It is presented as a percentage of a reference dose, which enables us to calculate the maximum acceptable daily dose. Classically, it is calculated as follows [59]:

2 $$\mathrm{D}=\frac{100}{{T}_{c}}{T}_{i}{10}^{\left(\frac{{L}_{i}-{L}_{c}}{q}\right)}$$

where *D* is the total noise dose (in percentage), *T*_*c*_ is the criterion sound duration (e.g., 24 h), *T*_*i*_ is the time exposure spent in the *ith* interval in hours, *L*_*c*_ is the criterion sound level (e.g., 59.3 dB(A)), *L*_*i*_ is the noise exposure intensity during the *ith* time interval, and *q* is the exchange rate parameter (dB)) (e.g., 10 for an exchange rate of 3 dB). As an example, with the reference exposure set to 59.3 dB(A) during 24 h, an exposure of 56.3 dB(A) during 24 h corresponds to a 50% dose. With the same reference, an exposure to 75 dB(A) during 40 min corresponds to a 103% dose.

Finally, in the same way as intake risk doses, the results of the GAMMAR model for noise exposure are used to predict how many minutes it takes to reach the dose corresponding to the daily mean exposure value of 59.3 dB (*L*_den_).

## Results

### Descriptive statistics: levels of exposure and inhalation

#### Levels of exposure and inhalation

The noise levels recorded during trips were particularly high with an overall noise mean of 79.7 dB(A) (Table 1). During 32% of the cycling time, the noise level exceeds 80 dB(A). Not surprisingly, exposure to NO_2_ and its inhalation vary significantly, with mean and standard deviation values of 201 µg/m^3^ and 79.4, and 7.1 µg and 4.3, respectively. Also, the three indicators are characterized by strong temporal and spatial autocorrelation (see ACF and Moran I values, Table 1).

**Table 1:** Descriptive statistics

Statistics	dB(A) (*L*_Aeq,1 min_)	NO_2_(µg/m^3^)	Inhalation (µg NO_2_)
N	4157	4368	4368
Percentiles			
1	68.1	29.1	0.8
5	71.7	83.8	2.2
10	73.1	109.5	3.0
25 (first quartile)	75.8	148.4	4.3
50 (median)	78.2	196.1	6.2
75 (third quartile)	80.7	245.5	9.3
90	82.8	296.7	13.1
95	84.3	340.5	15.4
99	86.6	431.0	21.2
Mean^a^	79.7	201.4	7.1
Standard deviation (SD)^a^	4.2	79.4	4.3
ACF^b^ index with			
k = 1	0.62	0.59	0.79
k = 2	0.35	0.38	0.64
Moran I^c^	0.36 (d = 250)	0.27 (d = 300)	0.29 (d = 450)

^a^Mean and SD of dB values are computed using the *seewave* package [60]. ^b^Autocorrelation function (ACF) index [43]. ^c^To calculate Moran’s I statistic, we used a binary matrix and defined as neighbours of the segment *i* all the segments in a buffer of length *d* around *i* with *d* ranging from 50 to 500 m with a step of 50 m. Only the highest values are reported here

#### Weather conditions

Throughout the data collection, we experienced steady weather conditions. The air humidity was moderate with a mean relative humidity of 46% (34%–60% for 5th and 95th percentile), and the average temperature was 29 °C (26–31 °C). During 36% of the data collection, the wind speed was 0 km/h. When wind was blowing, its mean speed was 9 km/h (4–11 km/h) with a dominant north-west direction. These conditions are typical of the weather in Delhi during this period of the year and they contribute to maintaining high levels of air pollution because of stagnant wind, the absence of rainfall, nocturnal radiative inversions and smoke blowing in from numerous agricultural fires [61, 62].

### GAMMAR models: factors associated with exposure and inhalation

#### Assessment of the models

All models′ parameters converged (Rhat = 1.0) and all the trace plots display important mixing (four chains) (Additional file 1: Figures S1, S2 and S3). The posterior predictive checks demonstrate that the three models are well fitted (Additional file 1: Figure S4). Also, the autocorrelation function (ACF) values calculated on the residuals suggest there is no temporal dependency in the three models (Additional file 1: Table S2).

The obtained Bayes R-squared are relatively similar among the three models when keeping only the fixed effects: 0.480, 0.397 and 0.419 for NO_2_ inhalation, NO_2_ exposure and noise exposure, respectively. However, they are higher for the inhalation and NO_2_ exposure models than for the noise exposure model with the random effects (0.650, 0.516, and 0.431, respectively). This implies that random effects play an important role in the NO_2_ model (effect of the Aeroqual monitor assigned to each participant) and inhalation model (combined effect of the Aeroqual monitor and the participants' physiological characteristics, i.e. participants’ different ventilation rates). Conversely, due to the higher accuracy of the dosimeters (Class 1), random effects play a minor role in the noise exposure prediction.

#### Analysis of the random linear effects

As a result, the intercepts of the random effects vary significantly according to the day of the week and the participant for the NO_2_ model, and only according to the participant for the inhalation model. Yet the day of the week has no significant impact on the noise exposure. This means that the noise levels measured are broadly similar for the five days of collection. However, these results demonstrate the relevance of introducing the day of the week and the participant as random effects in order to obtain unbiased coefficients for the fixed effects predictors.

#### Analysis of the fixed linear effects

Table 2 (fixed terms) presents the results of the predictors linearly added to the models. Concerning the weather conditions, the humidity has a positive impact on the levels of exposure and inhalation for the NO_2_. Because the wind disperses the air pollutants, it significantly reduces the cyclists' exposure to nitrogen dioxide and its inhalation by − 1.6 µg/m^3^ and − 0.1 µg for each km/h. The number of intersections crossed by the cyclist during a one-minute segment is weakly associated with a decrease of the three dependent variables. Second, the average speed tends to reduce the exposure levels to noise and air pollution, and the opposite is true for the NO_2_ inhaled dose. This is to be expected, as the faster cyclists go, the more they ventilate and the more they inhale air pollutants.

**Table 2:** Results of the Bayesian models

	dB(A) (*L*_Aeq,1 min_)		NO_2_ (µg/m^3^)		Inhalation (µg NO_2_)	
	Est	95% CI	Est	95% CI	Est	95% CI
Fixed effects						
(Intercept)	76.51	[74.11 78.06]	115.62	[77.90 152.81]	1.73	[− 4.02 7.35]
Humidity (%)	–	–	2.13	[1.56 2.70]	0.11	[0.08 0.17]
Wind speed (km/h)	–	–	− 1.60	[− 2.34 − 0.86]	− 0.09	− 0.13 − 0.05]
Intersections crossed (N)	− 0.05	[− 0.12 0.02]	− 1.84	[− 2.73 − 0.94]	− 0.02	[− 0.06 0.02]
Speed (km/h)	− 0.02	[− 0.03 − 0.00]	− 1.65	[− 1.87 − 1.44]	0.02	[0.01 0.03]
Slope (%)	0.00	[− 0.03 0.04]	− 0.25	[− 0.82 0.32]	− 0.01	[− 0.03 0.02]
Residential street	Ref		Ref		Ref	
Trunk or motorway	4.09	[3.02 5.14]	56.31	[40.55 72.22]	1.82	[0.96 2.71]
Primary road	3.35	[2.70 4.00]	47.21	[38.96 55.39]	1.28	[0.83 1.73]
Secondary road	2.42	[1.98 2.86]	36.77	[31.29 42.44]	1.01	[0.72 1.31]
Tertiary road	1.49	[0.96 1.82]	28.64	[23.64 33.76]	0.75	[0.49 1.02]
Unclassified or service road	0.75	[0.07 1.42]	3.10	[− 5.10 11.40]	0.07	[− 0.36 0.51]
Random effects (intercept)						
Monday	0.01	[− 0.35 0.40]	− 5.32	[− 21.57 11.15]	0.04	[− 1.00 1.10]
Tuesday	0.09	[− 0.23 0.56]	22.80	[7.08 40.01]	0.84	[− 0.19 1.96]
Wednesday	0.10	[− 0.22 0.55]	− 19.82	[− 36.15 − 3.47]	− 1.01	[− 2.09 0.04]
Friday	− 0.07	[− 0.48 0.27]	− 3.56	[− 19.65 13.02]	0.08	− 0.96 1.14]
Saturday	− 0.10	[− 0.56 0.23]	9.21	[− 6.90 26.12]	0.06	[− 1.00 1.14]
Participant I	0.68	[− 0.82 3.11]	24.80	[− 1.60 52.70]	3.30	[− 2.17 8.74]
Participant II	− 0.34	[− 1.82 2.05]	− 43.77	[− 70.17 − 16.08]	− 2.34	[− 7.77 3.10]
Participant III	0.40	[− 1.08 2.80]	36.25	[9.36 64.54]	− 0.80	[− 6.23 4.60]
Moving average						
MA [1]	0.61	[0.57 0.64]	0.57	[0.54 0.59]	0.68	[0.65 0.71]
MA [2]	0.20	[0.16 0.23]	0.18	[0.15 0.21]	0.31	[0.27 0.34]
MA [3]	0.08	[0.05 0.11]	0.08	[0.06 0.10]	0.14	[0.11 0.16]
Bayes marginal R-squared	0.419	[0.401 0.436]	0.397	[0.381 0.413]	0.480	[0.459 0.501]
Bayes conditional R-squared	0.431	[0.414 0.447]	0.516	[0.502 0.524]	0.650	[0.641 0.658]
Waic	20,628		46,503		19,438	

Third, the exposure to road traffic noise and air pollution and the inhaled dose of NO_2_ are strongly associated with the type of road taken by the cyclist. For example, compared to a residential street, spending one minute on a motorway or a trunk increases mean noise by 4.1 dB(A), mean value of NO_2_ by 56.3 µg/m^3^ and mean value of inhaled dose by 1.8 µg. Not surprisingly, these increases are lower for primary (3.4 dB(A), 47.2 µg/m^3^, 1.3 µg), secondary (2.4 dB(A), 36.8 µg/m^3^, 1.0 µg), tertiary (1.5 dB(A), 28.6 µg/m^3^, 0.8 µg), and unclassified roads (0.8 dB(A), 3.1 µg/m^3^, 0.1 µg). In short, the results of these three models confirm that the type of road taken by cyclists has an important impact on their exposure to air pollution, noise and inhalation of nitrogen dioxide.

#### Analysis of the non-linear effects

**Fig. 5:**
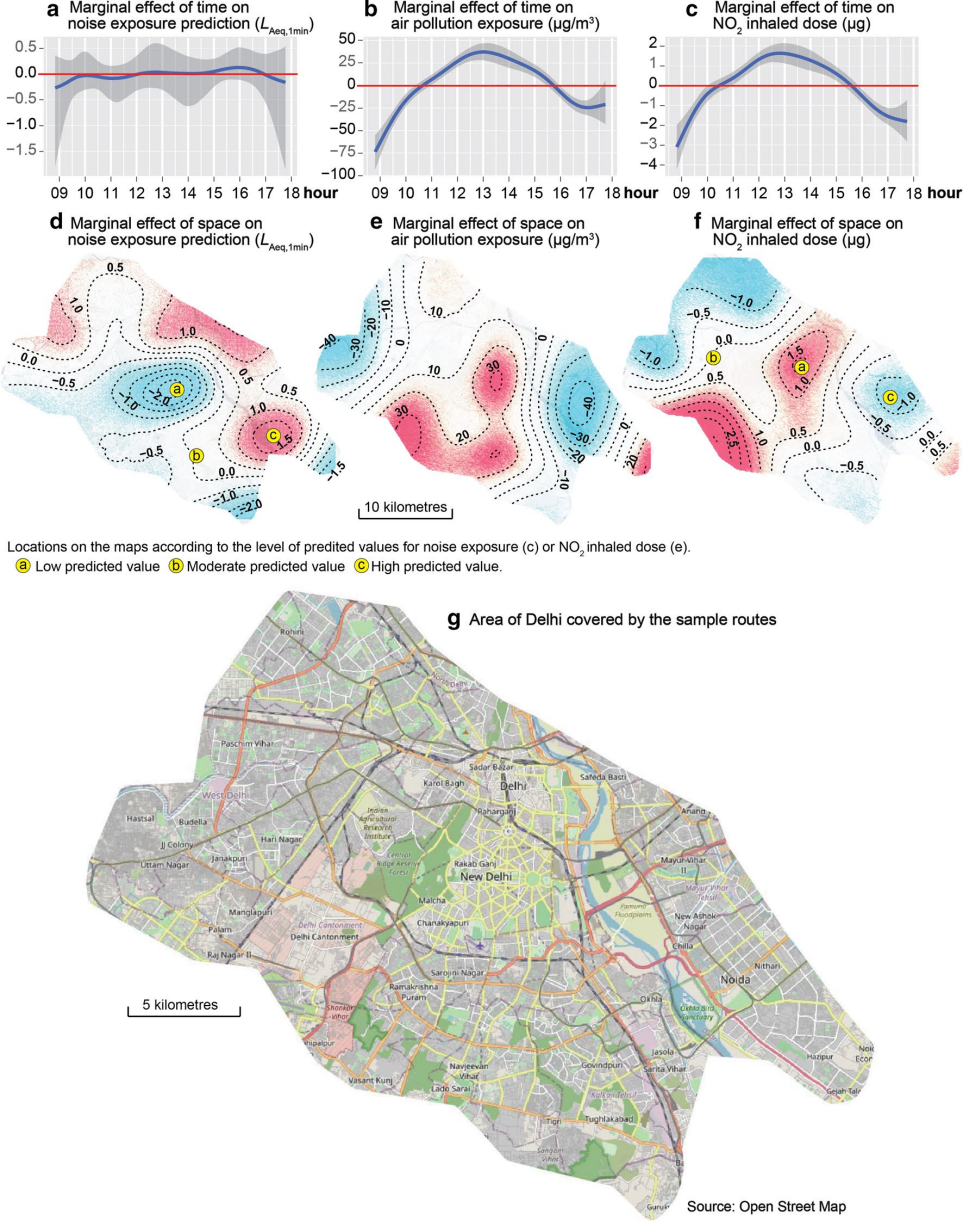
Marginal effects of time and space on the three dependent variable predictions

Figure 5 displays the marginal effects of time and space introduced as splines in our three models. For the temporal trends, the red horizontal lines plotted in Fig. 5a–c. represent the daily centred mean and allow us to note when the exposure and inhalation values are highest and lowest during the day, all else being equal. Concerning the temporal trend for noise exposure, the effect is not significant. This means that the cyclist’s exposure to road traffic noise is constantly high throughout the day in Delhi. By contrast, there is an important effect for the NO_2_ exposure and inhalation (about 100 µg/m^3^ and 5 µg, respectively), characterized by an increase in values during the morning, reaching a peak at 01:00 p.m., and a decrease during the afternoon.

Concerning the spatial patterns, the extent of the effect between the areas with the highest and lowest concentration levels reaches 4.5 dB(A), 70 µg/m^3^ and 2 µg for road traffic noise exposure, pollution exposure and the inhaled dose of NO_2_, respectively (Fig. 5c–e). The spatial trend for noise is quite different for that of the other two. Overall, the central part of Delhi has the lowest noise levels, but the highest exposure and inhaled dose levels. However, these temporal and spatial trends could be characterized by strong variability and uncertainty, and thus must be interpreted with caution. These spatial and temporal trends are valid only for the data collection period; they cannot be generalized over the whole year. Nevertheless, this underlines the need to control the spatial and temporal effects in order to obtain more robust coefficients for the fixed effects predictors.

### Potential adverse health effects of road traffic noise and air pollution

**Fig. 6:**
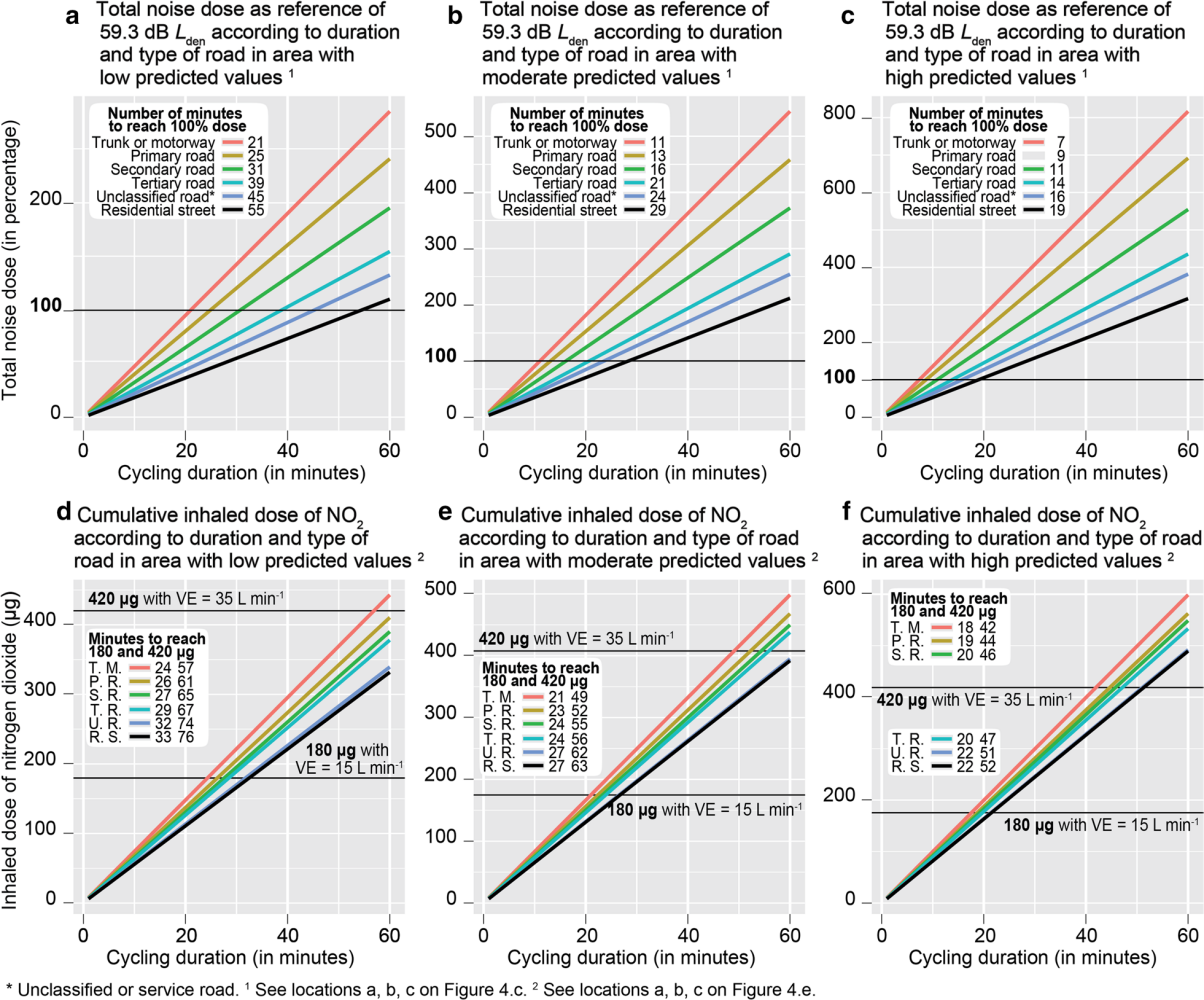
Noise dose and inhalation dose of nitrogen dioxide and hazard

As mentioned before, three guideline values were selected to assess potential adverse health effects: 59.3 dB *L*_den_ for noise and 180 µg and 420 µg, as inhaled doses of NO_2_ for a pedestrian and a cyclist, respectively. We previously demonstrated that levels of noise and air pollution exposure vary substantially throughout Delhi, as in most cities, and according to the type of road taken by the cyclist. Consequently, we propose here to estimate after how many minutes a cyclist would accumulate a dose above the aforementioned thresholds, for three areas in Delhi (see Fig. 6) where levels of noise and air pollution were low, moderate and high during our data collection, all else being equal. To do so, we predict values of the noise dose and the inhaled NO_2_ dose for these three locations and the road type by using the Bayesian models’ results. Note that for these predictions, mean values were set for several independent variables (humidity, wind speed, and slope), the time was set at 11:00 a.m., and the geographic coordinates (x,y) of each location were specified. The random effects were not included in predictions to avoid overestimation. The results are plotted in Fig. 6.

For noise exposure, the equivalent of a daily mean exposure of 59.9 dB *L*_den_ is reached quickly, regardless of road type and location. For example, during the data collection, if we had only cycled on residential streets, we would have reached the noise risk cutpoint after 55, 29 and 19 min, in areas with low, moderate and high noise exposure values respectively (Fig. 6a–c). In the same way, for primary roads, only 25, 13 and 9 min would have been needed.

For the nitrogen dioxide, the cumulative intake risk value for one hour is also reached quickly with a pedestrian’s ventilation rate (180 µg with 15 L/min) and, to a lesser extent, for a cyclist’s ventilation rate (420 µg with 35 L/min). For example, if we had only cycled on residential streets, we would have reached the intake risk cutpoints after 33, 27 and 22 min for 180 µg and 76, 63 and 52 min for 420 µg in areas with low, moderate and high air pollution exposure values respectively (Fig. 6d–f). For primary roads, only 26, 23 and 19 min for 180 µg and 53, 46 and 40 for 420 µg would have been needed.

The results demonstrate that the type of road taken by the cyclist has a huge impact on the health hazard. By way of example, after one hour cycling in an area with moderate predicted values of noise and air pollution, the noise dose and inhaled dose of NO_2_ will reach 212% and 403 µg respectively on residential streets, and 459% and 482 µg on primary roads.

## Discussion

### Research limitations

In this study, low-cost air quality monitors were used to investigate cyclists’ exposure to air pollution. This practice is not new and takes root in the development of a new paradigm around the measure of air pollution [63]. Mobile data collection with low-cost sensors has many recognized advantages (finer space–time coverage and better approximation of individual exposure levels) but also several drawbacks (lower accuracy and small-time coverage). It is generally agreed that this type of work produces complementary data and knowledge to the traditional reference monitoring networks [64]. In our case, two main concerns should be raised.

First, the Aeroqual NO_2_ sensor is known for its cross-sensitivity to ozone (O_3_) [65] which could lead to an overestimation of NO_2_ exposure. It must be pointed out that O_3_ is another potentially harmful pollutant which moderates this problem of overestimation. However, O_3_ is known to be a secondary pollutant following specific daily patterns in Delhi [61]. The temporal splines in our models of NO_2_ exposure and inhalation follow this same trend with a similar effect size. That means its contribution in the models is controlled by the temporal splines Second, systematic differences across sensors could occur but have been controlled here with random effects. Consequently, temporal spline and random effects allow us to obtain unbiased coefficients for the fixed effects. The second limitation is the absence of a sensor to measure particulate matter, especially PM_2.5_. This pollutant is the most investigated in this research area owing to its well-established impact on health [21]. Consequently, future works should be conducted using multiple sensors (e.g. NO_2_, O_3_, PM_2.5_ and PM_10_).

### Health equity issues

Air pollution and noise exposure compound the hostile conditions deterring bicycle use [3]. Therefore, cycling in Delhi is, simultaneously, an issue of urban transport justice [66], environmental equity [67] and gender equity [68].

First, cycling in Delhi is a clear situation of urban transport injustice [66]. While cycling is deemed to be among the most sustainable transport modes, cyclists receive considerably less political attention and infrastructure investment, and are exposed to harmful pollutants that they do not produce [26].

Second, in Delhi most cyclists are low-income individuals, in particular informal and casual workers, for whom cycling is the only alternative [7]. They may experience significant adverse effects of simultaneous air pollution and noise exposure while lacking sufficient financial resources to afford healthcare. For example, a qualitative study on noise pollution in Delhi (a field survey of 1,693 households) found noise pollution causes significant perceived adverse health effects, such as annoyance (28.16%), interference with communication (27.15%) and headache/nausea/giddiness/fatigue (26.43%) [69].

Third, amongst the most disadvantaged people of Delhi, women face additional challenges, as a result of their household responsibilities (taking children to school, visiting elderly relatives, shopping for groceries, etc.), differentiated travel needs and capacities (i.e. less control over the financial and mobility resources of the household) and greater vulnerability and sensitivity to harsh road environments (inadequate pavement quality and lane width, harassment, etc.). These are barriers to using a bicycle; but at the same time, using a bicycle can become a way of improving women's condition, allowing them to move more independently throughout the day while carrying children and goods, and accessing more diverse work opportunities [3].

All in all, improving general cycling conditions is a major health issue which Delhi transport planners must face in order to mitigate these (sometimes cumulative) iniquities [70].

### Implications for policy makers

Translating our results into specific policy recommendations is challenging, since most research about cycling in cities is conducted in Global North settings. While our research brings significant contributions to the field of cyclists' exposure to noise and air pollution in a Global South city (Delhi), more research is needed to understand how Delhi policy makers could effectively tackle cyclists' exposure to noise and NO_2_. Meanwhile, one must refrain from directly applying city cycling knowledge from Global North to Global South cities, to avoid unintended outcomes [71]. For example, Agarwal and Kaddoura [72] estimated that building a proposed bicycle superhighway in Patna, India would increase cyclists’ exposure to air pollution, due to the anticipated congestion on the cycle track and consequent higher travel time in heavily polluted environments. Conversely, results from Bangalore, India suggest that providing more non-motorized transport infrastructure (footpaths and cycle-lanes) in the Central Business District would improve many sustainability indicators (including noise and air pollution), and would benefit in priority to low-income population groups [73]. These conflicting results underline the need for context-specific and evidence-based policy and infrastructure planning, and calls for more research in Global South contexts. The results presented here could be used by policy makers during their planning process to compare the potential exposure to air and noise pollution induced by foreseen infrastructures.

## Conclusion

The results of this study reveal significant variations in cyclists’ exposure to noise and air pollution from one road type to another. We calculated that during our study period, depending on the spatial location and type of road taken, cyclists often reached (and exceeded) the estimated hazardous dose thresholds of both noise and nitrogen dioxide in less than an hour. For noise, the equivalent of a daily mean exposure of 59.9 dB *L*_den_ is reached quickly: 29, 21 and 13 min on a residential street, tertiary road and primary road in an area with moderate predicted values of noise. In the same way, the cumulative NO_2_ intake risk value for one hour is also reached quickly: 27, 24, 23 min on a residential street, tertiary road and primary road. Such findings are particularly worrisome, since they add to the numerous perils that cyclists–a very deprived population in Delhi–already face. Future research could concentrate on simultaneously measuring several air pollutants (e.g. PM_2.5_, NO_2_ and O_3_) and noise, since they are also known to have adverse health effects and to be present in high levels in many Global South cities. Participative approaches could be helpful gaining an understanding of how cyclists feel in and adapt to such hostile road environments.

## Supplementary Information

**Additional file 1.** Supplementary Material.

## Data Availability

Please contact author for data requests.

## Abbreviations

dB(A): A-weighted decibel
GAMMAR: Generalized additive mixed models with auto-regressive terms
*L*_Aeq,1min_: Energy equivalent sound pressure level in dB(A) over one minute
*L*_den_: The A-weighted, Leq (equivalent noise level) over a whole day
NO_2_: Nitrogen dioxide
O_3_: Ozone
OSM: Open Street Map
PM_2.5_: Fine Particulate Matter with a diameter of 2.5 micrometres (μm) or less
PM_10_: Coarse Particulate Matter with a diameter of 10 micrometres (μm) or less
WHO: World Health Organization
