# Supplementary material for: Cycling in one of the most polluted cities in the world: Exposure to noise and air pollution and potential adverse health impacts in Delhi
Source: Int J Health Geogr. 2021 Apr 30;20:18. doi: 10.1186/s12942-021-00272-2 (PMC8086121; doi:10.1186/s12942-021-00272-2)
Supplement: Supplementary file 1 — Additional file 1. Supplementary Material. [file 12942_2021_272_MOESM1_ESM.docx]

Appendix A: Supplementary Material

Description of Priors Used in the Models

All the priors are of a type “weakly informative” and conservative (Table S1). This is justified by the lack of information about expected values and by the fairly large amount of data. consequently, strong priors are not required. For the random effects, we followed the recommendation of Gelman [1] and used proper priors with a half-Cauchy distribution. In *brms*, spline variability is modelled as a random effect due to proximity between them. Therefore, the same types of prior are used (half-Cauchy). The scale parameters for time and space splines are 10 and 20 respectively. This reflects the prior belief that we expect more variation caused by space than by the time of the day and the day of the week.

[1] Gelman, A. (2006). "Prior distributions for variance parameters in hierarchical models (comment on article by Browne and Draper)." Bayesian analysis 1(3): 515-534.

**Table S1.** Priors for the three Bayesian models.

| Variable | dB(A) (*L*_Aeq,1min_) | NO_2_  (µg/m^3^) | Inhalation  (µg NO_2_) |  |
| --- | --- | --- | --- | --- |
| Fixed effects |  |  |  |  |
| Humidity (%) | -- | normal(0,10) | normal(0,2) | |
| Wind speed (km/h) | -- | normal(0,10) | normal(0,2) | |
| Intersections crossed (N) | normal(0,3) | normal(0,10) | normal(0,2) | |
| Speed (km/h) | normal(0,3) | normal(0,10) | normal(0,2) | |
| Slope (%) | normal(0,3) | normal(0,10) | normal(0,2) | |
| Trunk or motorway | normal(3,3) | normal(30,15) | normal(0.70,2) | |
| Primary road | normal(2,3) | normal(20,15) | normal(0.50,2) | |
| Secondary road | normal(1,3) | normal(15,15) | normal(0.35,2) | |
| Tertiary road | normal(0,3) | normal(5,10) | normal(0.10,2) | |
| Unclassified or service road | normal(0,3) | normal(0,10) | normal(0.0,2) | |
| Random effects (intercept) |  |  |  | |
| Weekday | cauchy(0,5) | cauchy(0,5) | cauchy(0,5) | |
| Participant | cauchy(0,5) | cauchy(0,5) | cauchy(0,5) | |
| Non-linear effects |  |  |  | |
| Temporal spline | cauchy(0,10) | cauchy(0,10) | cauchy(0,10) | |
| Spatial spline | cauchy(0,20) | cauchy(0,20) | cauchy(0,20) | |

**Table S2.** Temporal dependency in the residuals for three Bayesian models (ACF).

| Lag | dB(A)  (*L*_Aeq,1min_) | NO_2_  (µg/m^3^) | Inhalation  (µg NO_2_) |
| --- | --- | --- | --- |
| 2 | −0.029 | −0.105 | −0.044 |
| 3 | −0.001 | −0.045 | −-0.008 |
| 4 | 0.031 | 0.010 | 0.081 |

R Code example

library("brms")

#---- Bayesian model of NO2 ------------

# Defining the priors

PriorsNO2 <-

c(

# Priors for fixed effects

set_prior("normal(200,15)", class="Intercept",coef=""),

set_prior("normal(0,10)", class="b", coef="RH"),

set_prior("normal(0,10)", class="b", coef="WindSpeed"),

set_prior("normal(0,10)", class="b", coef="NbInter"),

set_prior("normal(0,10)", class="b", coef="SpeedKmh"),

set_prior("normal(0,10)", class="b", coef="Slope"),

set_prior("normal(30,15)", class="b", coef="H_trunk_motorway"),

set_prior("normal(20,15)", class="b", coef="H_primary"),

set_prior("normal(15,15)", class="b", coef="H_secondar"),

set_prior("normal(5,10)", class="b", coef="H_tertiary"),

set_prior("normal(0,10)", class="b", coef="H_unclassi_None_service"),

# Priors for the nonlinear effects (splines)

set_prior("cauchy(0,10)", class="sds", coef="s(PassedMin)"),

set_prior("cauchy(0,20)", class="sds", coef='s(X_7760,Y_7760)'),

# Priors for random fixed effects

set_prior("cauchy(0,5)", class="sd", group="WeekDay"),

set_prior("cauchy(0,5)", class="sd", group="IDPart")

)

# Building the model

BayesNO2_Tscaled <- brm(NO2ugm ~

# Fixed effects

RH+WindSpeed+

NbInter+SpeedKmh+Slope+

H_trunk_motorway+H_primary+H_secondar+

H_tertiary+H_unclassi_None_service+

# Nonlinear effects (splines)

s(PassedMin)+s(X_7760,Y_7760)+

# Random effects

(1|WeekDay)+(1|IDPart),

# moving average (MA) structure of arbitrary order

autocor=cor_ma(formula = ~ OID|IDTraj,q=3),

family=student(link = "identity"), data=AllDatasFinal_NO2,

prior = PriorsNO2,

chains=4, cores=4, seed = 13,

warmup=1000, iter = 4000,

control = list(adapt_delta = 0.99,max_treedepth=15))

# Results of the Model

summary(BayesDBA_TScaled) # Model summary

ranef(BayesDBA_TScaled) # Random coefficient

bayes_R2(BayesDBA_TScaled, loo=TRUE, re_formula = NA) # Marginal R2

bayes_R2(BayesDBA_TScaled, loo=TRUE) # R2 conditionnel # Conditionnal R2

waic(BayesDBA_TScaled)


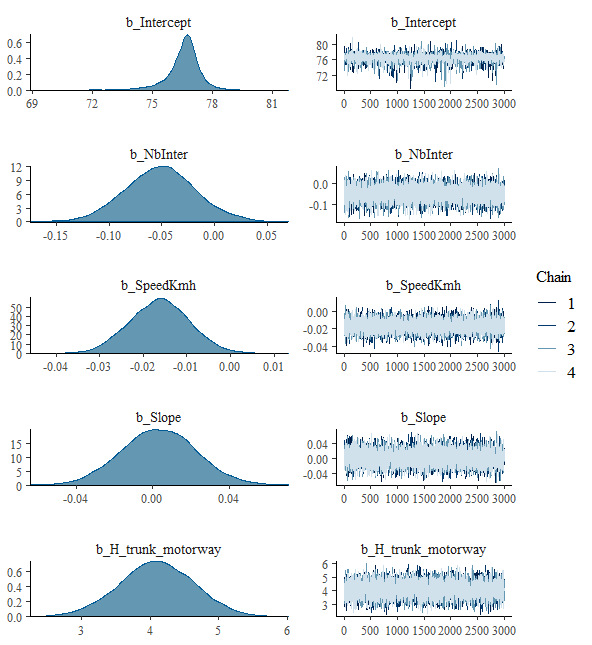


**Figure S1.** Posterior distributions and mixing chains for noise exposure model parameters.


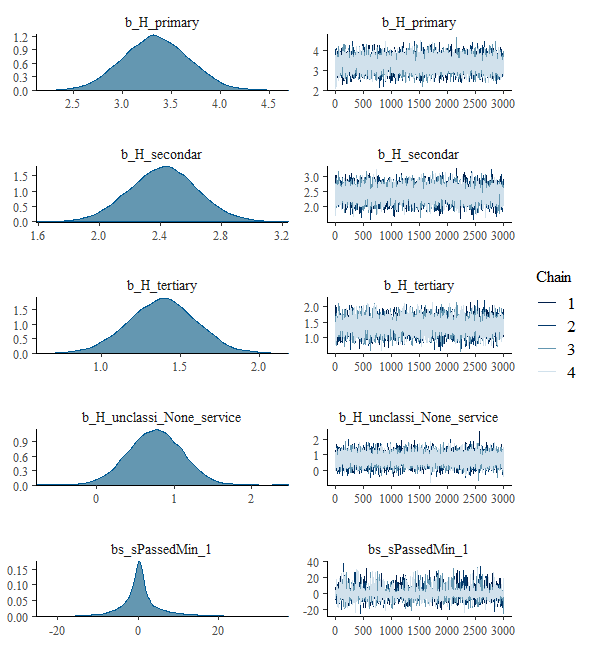


**Figure S1.** Posterior distributions and mixing chains for noise exposure model parameters (continued).


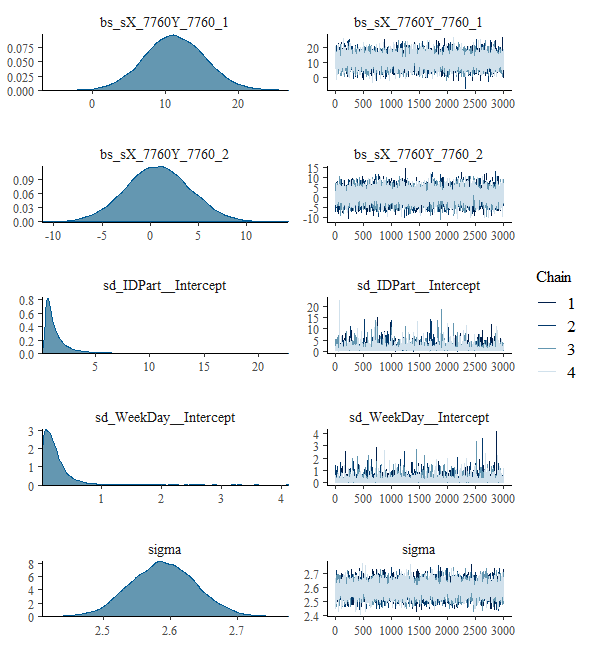


**Figure S1.** Posterior distributions and mixing chains for noise exposure model parameters (continued).


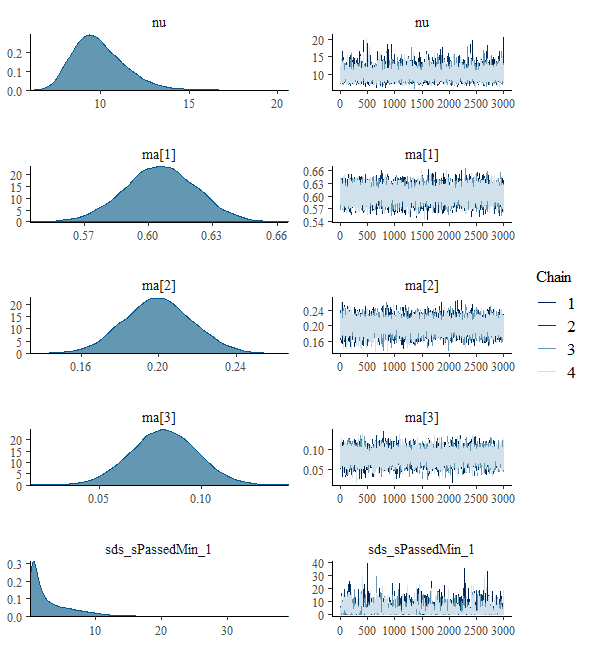


**Figure S1.** Posterior distributions and mixing chains for noise exposure model parameters (continued).


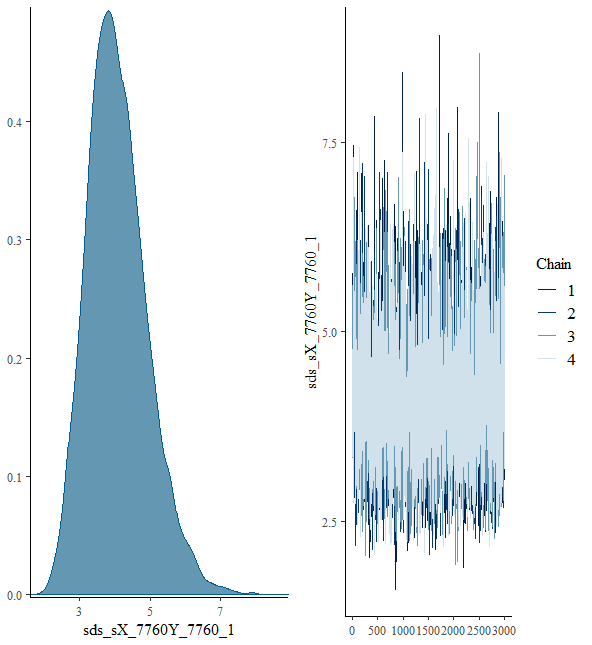


**Figure S1.** Posterior distributions and mixing chains for noise exposure model parameters (continued).


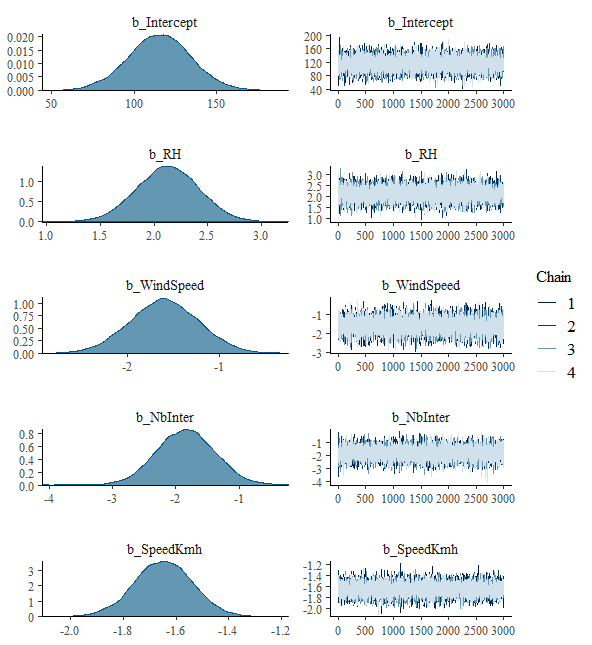


**Figure S2.** Posterior distributions and mixing chains for NO_2_ exposure model parameters.


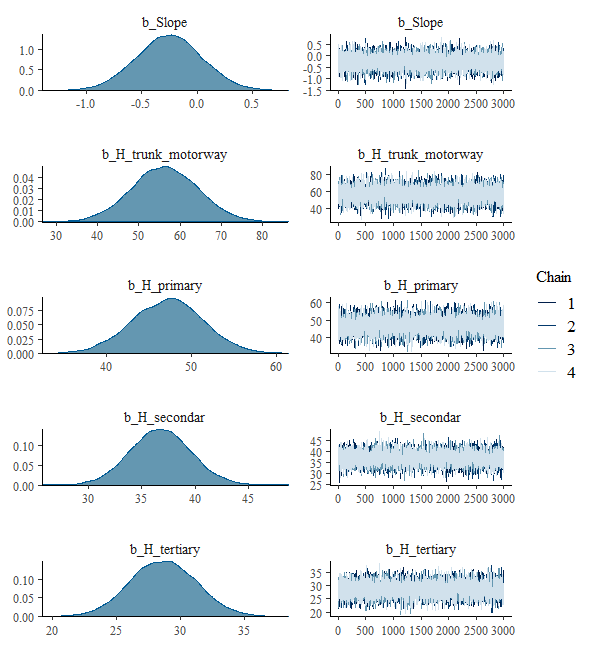


**Figure S2.** Posterior distributions and mixing chains for NO_2_ exposure model parameters(continued).


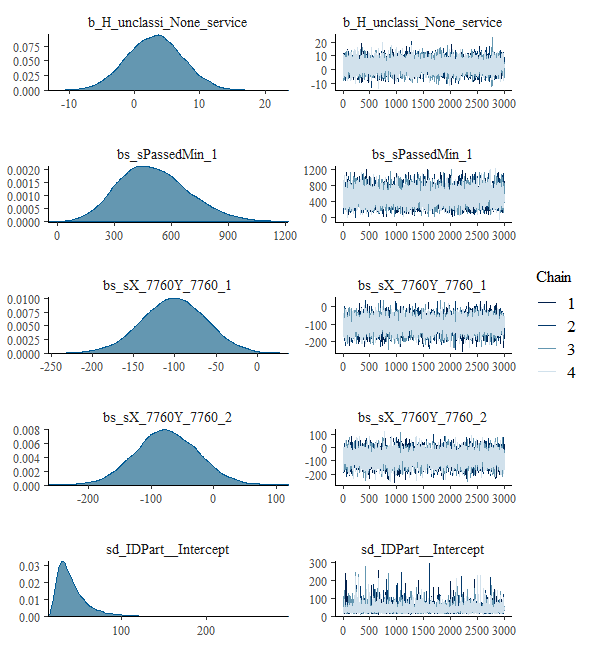


**Figure S2.** Posterior distributions and mixing chains for NO_2_ exposure model parameters(continued).


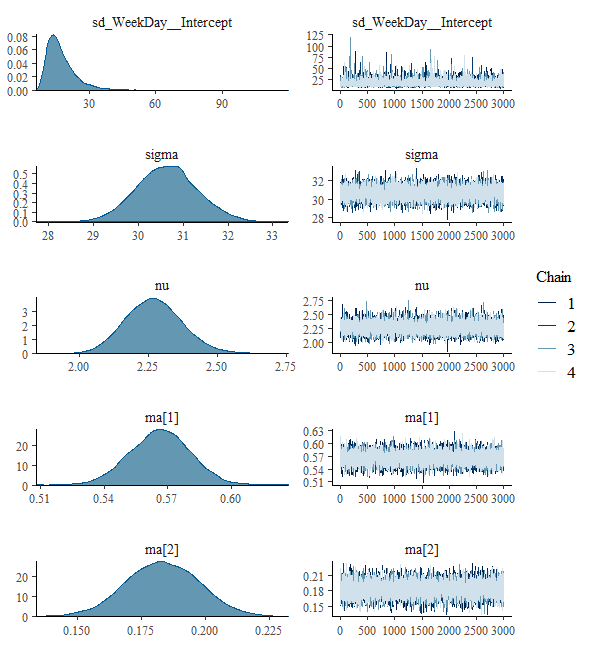


**Figure S2.** Posterior distributions and mixing chains for NO_2_ exposure model parameters(continued).


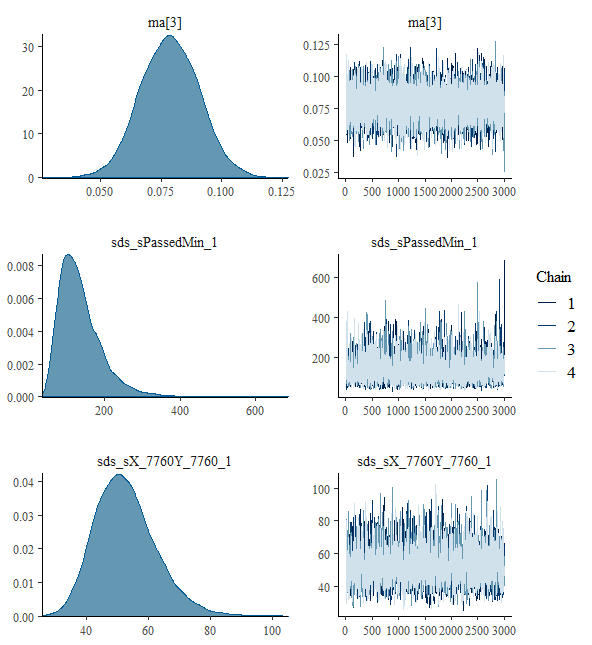


**Figure S2.** Posterior distributions and mixing chains for NO_2_ exposure model parameters(continued).


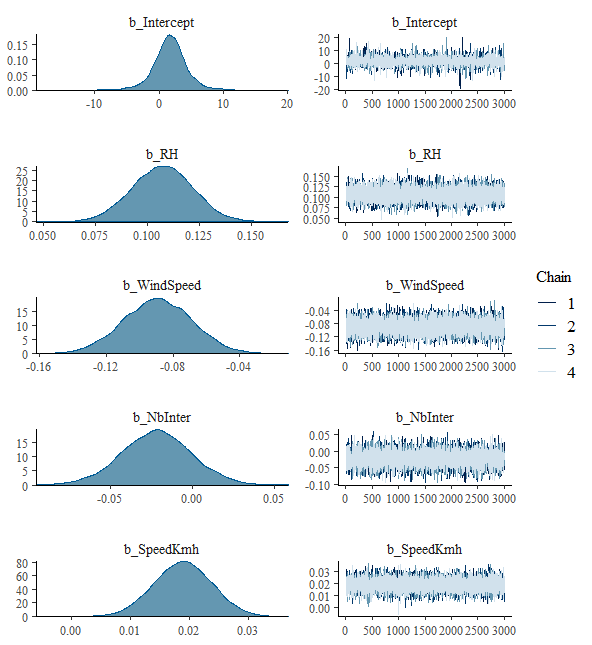


**Figure S3.** Posterior distributions and mixing chains for Montreal NO_2_ inhalation model parameters.


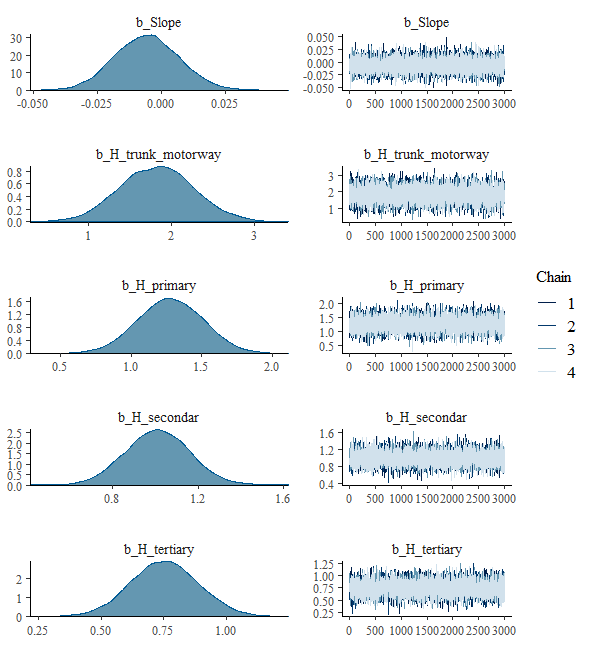


**Figure S3.** Posterior distributions and mixing chains for Montreal NO_2_ inhalation model parameters (continued).


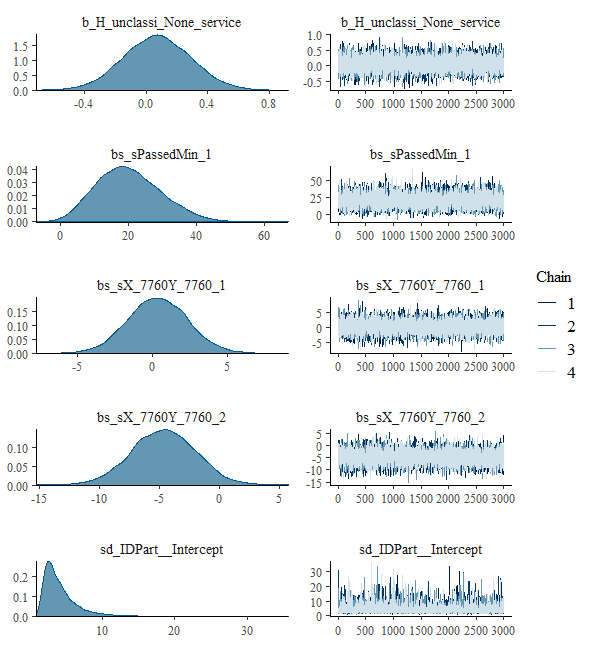


**Figure S3.** Posterior distributions and mixing chains for Montreal NO_2_ inhalation model parameters (continued).


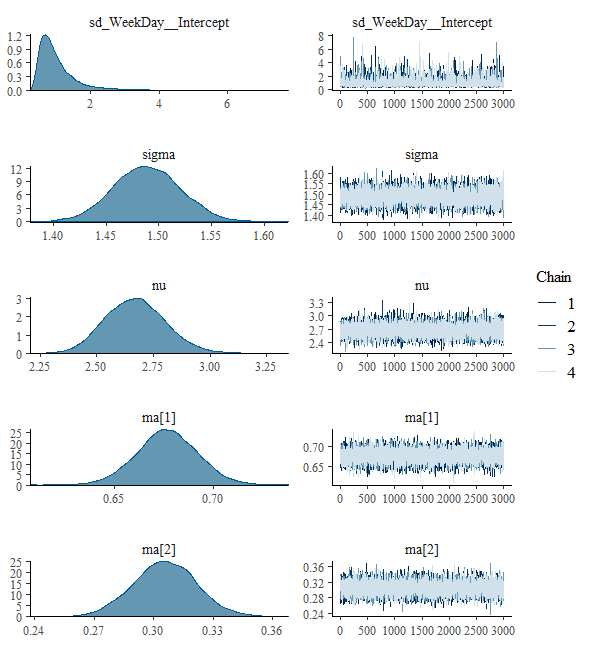


**Figure S3.** Posterior distributions and mixing chains for Montreal NO_2_ inhalation model parameters (continued).


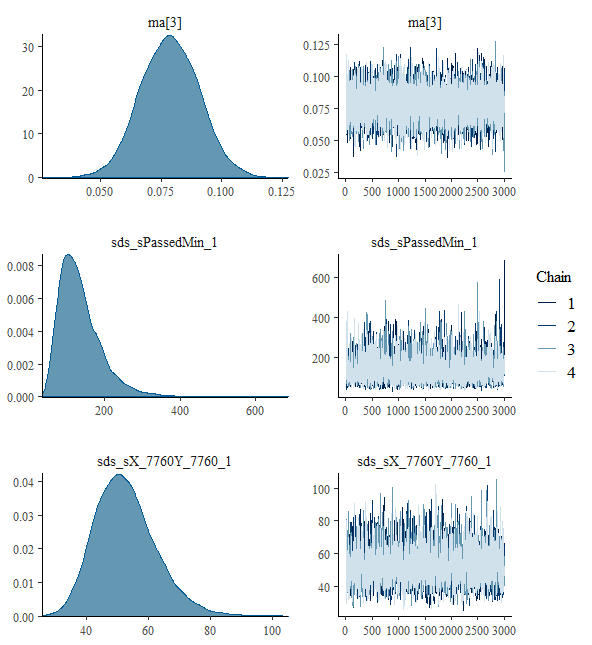


**Figure S3.** Posterior distributions and mixing chains for Montreal NO_2_ inhalation model parameters (continued).


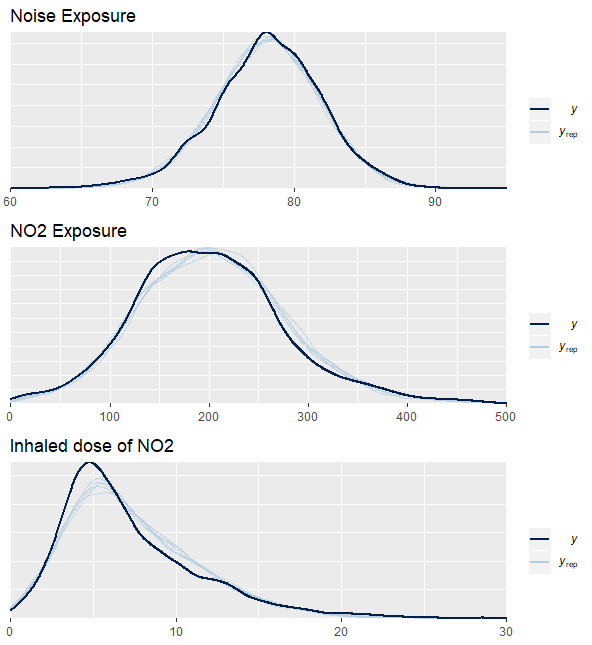


**Figure S4.** Posterior predictive checks for the three models (density overlay plot).
